# Characterization and Comparison of Postnatal Rat Meniscus Stem Cells at Different Developmental Stages

**DOI:** 10.1002/sctm.19-0125

**Published:** 2019-10-22

**Authors:** Shaoqi He, Dengfeng Ruan, Yangwu Chen, Jisheng Ran, Xiao Chen, Zi Yin, Chenqi Tang, Jiayun Huang, Boon Chin Heng, Jialin Chen, Weishan Chen, Weiliang Shen, Hongwei Ouyang

**Affiliations:** ^1^ Department of Orthopedic Surgery The Second Affiliated Hospital, Zhejiang University School of Medicine Hangzhou People's Republic of China; ^2^ Department of Orthopedic Surgery Third Affiliated Hospital of Wenzhou Medical University Wenzhou People's Republic of China; ^3^ Department of Sports Medicine Zhejiang University School of Medicine Hangzhou People's Republic of China; ^4^ Peking University School of Stomatology Beijing People's Republic of China; ^5^ School of Medicine Southeast University Nanjing People's Republic of China; ^6^ Department of Orthopedics Research Institute of Zhejiang University Hangzhou People's Republic of China; ^7^ China Orthopaedic Regenerative Medicine (CORMed) Hangzhou People's Republic of China

**Keywords:** Developmental biology, Mesenchymal stem cells (MSCs), Stem cell transplantation, Tissue regeneration, Tissue‐specific stem cells

## Abstract

Meniscus‐derived stem cells (MeSCs) are a potential cell source for meniscus tissue engineering. The stark morphological and structural changes of meniscus tissue during development indicate the complexity of MeSCs at different tissue regions and stages of development. In this study, we characterized and compared postnatal rat meniscus tissue and MeSCs at different tissue regions and stages of development. We observed that the rat meniscus tissue exhibited marked changes in tissue morphology during development, with day 7 being the most representative time point of different developmental stages. All rat MeSCs displayed typical stem cell characteristics. Rat MeSCs derived from day 7 inner meniscus tissue exhibited the highest self‐renewal capacity, cell proliferation, differentiation potential toward various mesenchymal lineage and the highest expression levels of chondrogenic genes and proteins. Transplantation of rat MeSCs derived from day 7 inner meniscus tissue promoted neo‐tissue formation and effectively protected joint surface cartilage in vivo. Our results demonstrated for the first time that rat MeSCs are not necessarily better at earlier developmental stages, and that rat MeSCs derived from day 7 inner meniscus tissue may be a superior cell source for effective meniscus regeneration and articular cartilage protection. This information could make a significant contribution to human meniscus tissue engineering in the future. stem cells translational medicine *2019;8:1318&1329*

## Abstract 2

**(A):** Meniscus tissue at different tissue regions and stages of development. **(B):** MeSCs at different tissue regions and stages of development. **(C):** Intra‐articular injection of MeSCs for meniscus regeneration and OA suppression. *Significant difference between two groups at *p* < .05. **Significant difference between two groups at *p* < .01. ***Significant difference between two groups at *p* < .001. ****Significant difference between two groups at *p* < .0001. N.S., No significant difference between two groups at *p* ≥ .05.

## Graphical abstract

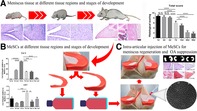

**Significance Statement**

The results of the study demonstrated for the first time that MeSCs are not necessarily better at earlier developmental stages, and that MeSCs derived from day 7 inner meniscus tissue may be a superior cell source for effective meniscus regeneration and articular cartilage protection. This information could make a significant contribution to human meniscus tissue engineering in the future.

## Introduction

Meniscus tears frequently occur during athletic injuries of the knee, disrupting the patient's mobility, and work capacity, as well as ruining the career of athletes 1. So, retaining, repairing, or even replacing the meniscus receives increasingly more attention. Injuries within the inner nonvascularized portion of the meniscus often have poor self‐regenerative capacity 1, 2, 3, 4. Meniscectomy is currently the most common treatment modality for meniscus tear 5. However, untreated meniscus tears or meniscectomy alters the loading environment in the knee joint, resulting in increased contact pressures and stresses on the articular cartilage, which is associated with the accelerated onset of long‐term degenerative joint changes, articular cartilage degeneration, and ultimately, osteoarthritis (OA) 3, 6, 7, 8. Hence, preservation and restoration of the damaged meniscus is necessary but remains an ongoing challenge 9.

Recently, mesenchymal stem cells (MSCs)‐based tissue‐engineering strategies have emerged to repair and facilitate regeneration of the injured meniscus. To date, various MSCs types have been proven beneficial for tissue engineering due to their evident chondrogenic differentiation potential, such as bone marrow‐derived MSCs (BMSCs), synovium‐derived MSCs (SMSCs), adipose‐derived MSCs, and blood vessel‐derived stem cells 1, 4, 10, 11. However, these cell sources are associated with donor site morbidity and ectopic bone formation 12, 13. Our team first proposed the concept of meniscus‐derived MSCs (MeSCs) that are present in the meniscus 14, 15. These cells possess self‐renewal capacity and multi‐differentiation potential and have immunosuppressive properties 14, 15, 16. The inherent capacity for expression of type II collagen by MeSCs is greater than that of BMSCs and SMSCs 17, 18, 19. Several studies have used MeSCs for meniscus tissue engineering, which has shown much potential for meniscus regeneration, thereby delaying or reducing the progression of OA induced by meniscectomy 14, 15, 18.

There are significant differences in meniscus tissue at different postnatal developmental stages 20, 21, 22, 23 or between inner and outer regions 24, indicating that cells isolated from different developmental stages or different tissue regions harbor different regeneration potential. Moreover, young and adult MSCs possess different repair capacities 25, 26, 27. The ideal seed cells for meniscus regeneration should express more chondrogenic and angiogenic genes and proteins. It has been reported that the expression of type II collagen and angiogenesis in meniscus tissue at different developmental stages are significantly different 28, 29. Correspondingly, the gene expression profiles of meniscus cells at different developmental stages are also significantly different 30.

Based on these previous studies, we hypothesize that there will be differences in the self‐renewal capacity and multilineage differentiation potential of MeSCs in different tissue regions and at different stages of development, which would in turn influence the capacity of these cells to contribute to meniscus regeneration and protection of articular cartilage. To test our hypothesis, this study will thus (a) characterize and compare postnatal rat meniscus tendon tissues at different tissue regions and stages of development by histological staining, polarized light microscopy and immunohistochemistry observations; (b) evaluate and compare the colony formation capacity, cell proliferation, surface CD marker expression, in vitro gene and protein expression profiles, as well as the differentiation potential of MeSCs localized in different tissue regions and at varying developmental stages; and (c) investigate and compare the regenerative capacity of these cells on meniscus repair and effects on the suppression of early experimental OA in vivo within a rat model.

## Materials and Methods

Female Sprague Dawley (SD) rats from different postnatal stages: 1, 2, 4, 7, 14, 28, and 56 days were used in the in vitro experiments. Another 20 female SD rats weighing 240–260 g were used in the in vivo study. Rat MeSCs derived from three different postnatal stages and two regions were used in all the experiments. Details are as follows: 1 day inner (MeSCs‐1d‐in) and 1day outer (MeSCs‐1d‐out), 7 days inner (MeSCs‐7d‐in) and 7 days outer (MeSCs‐7d‐out), 8 weeks inner (MeSCs‐8w‐in) and 8 weeks outer (MeSCs‐8w‐out).

### H&E Staining

Harvested specimens (*n* ≥ 3 at each time point) were fixed in 10% (v/v) neutral buffered formalin, decalcified with 10% ethylene diamine tetraacetic acid (EDTA), dehydrated through an alcohol gradient, cleared, and embedded within paraffin blocks. Histological sections (5 μm) were prepared using a microtome and subsequently deparaffinized with xylene, hydrated using decreasing concentrations of ethanol, and then subjected to hematoxylin and eosin (H&E) staining as described previously 31, prior to being observed under polarized light microscopy.

A blinded semiquantitative histological scoring system was a modification of the protocol utilized in previous studies 27, 32, 33. Three parameters (fiber structure, fibrocartilage structure, and cell number density) were semiquantitatively assessed (Supplementary Table S2).

### Immunohistochemistry

A series of 5‐μm‐thick sections were used for immunohistochemical staining according to established protocols 14. Rabbit anti‐rat polyclonal antibodies against collagen type II (Sigma‐Aldrich, St. Louis, MO, http://www.sigmaaldrich.com) together with goat anti‐mouse (Beyotime, Shanghai, China, https://www.beyotime.com) secondary antibodies were used to detect the expression of collagen type II within the meniscus.

### Low‐Density Seeding of MeSCs

The isolation and culture protocols for rat MeSCs were based on previous studies 14, 15. Rat meniscus tissues from three different postnatal stages: 1, 7, and 56 days were obtained. Each meniscus was separated into inner and outer regions under an operating microscope (SZX7, Olympus, Tokyo, Japan, https://www.olympus-global.com). The midline between the innermost and outermost sides of the meniscus was used as the boundary of inner and outer regions (Supplementary Fig. S1). Details are given in the Supplementary Materials.

### Colony‐Forming Assay

For the colony‐forming analysis, cells were harvested and plated onto a flask at a density of 300 cells per 25 cm^2^. The medium was replaced every 3 days. After incubation for 14 days, the colonies formed by MeSCs were fixed with 4% paraformaldehyde solution for 20 minutes and then stained with 1% (w/v) crystal violet (Sigma) in methanol for 15 minutes, and the number of colonies (with diameter >2 mm) were counted.

### FACS Analysis

MeSCs were fixed with 4% (w/v) paraformaldehyde in phosphate buffered solution (PBS) for 30 minutes at room temperature. Fixed cells (5 × 10^5^) in suspension were incubated with 1 mg of Fluorescein isothiocyanate (FITC)‐conjugated mouse anti‐rat monoclonal antibodies specific to CD29, CD44, CD45, CD34, CD90, and CD 105 (from BD Pharmingen, San Diego, CA, https://www.bdbiosciences.com; Santa Cruz Pharmingen, Santa Cruz, CA, https://www.scbt.com, respectively) for 1 hour at 4°C, and resuspended in 1 ml of PBS. For the isotype control, FITC‐coupled nonspecific mouse IgG (BD Pharmingen) was substituted for the primary antibody. Quantitative analysis was performed using FACS Canto II flow cytometer and FlowJo software.

### Cell Proliferation Assay

Cell proliferation was determined using the Cell Counting KIT‐8 (CCK‐8, Dojindo) according to the manufacturer's protocol. Detailed procedures are presented in the Supplementary Materials.

### Multipotent Differentiation Potential of MeSCs

The multipotent differentiation potential of MeSCs toward the adipogenic, osteogenic, and chondrogenic lineages was evaluated in vitro according to established protocols 14. Differentiation media were purchased from Cyagen Biosciences, Guangzhou, China. Positive induction of adipogenesis, osteogenesis, and chondrogenesis was confirmed by Oil Red O staining, alizarin red (ARS) and alkaline phosphatase (ALK) staining, and safranin O (S‐O) and Alcian blue staining, respectively 14, 15.

### RNA Isolation and Quantitative Real‐Time Polymerase Chain Reaction (qPCR) Analysis

RNA isolation and qPCR analysis of the expression of collagen II, aggrecan, Sox‐9, collagen I, MKX, and SCX genes were carried out according to established protocols 14, 15. For further details, see the Supplementary Materials. The sequences of primers used in this study are listed in Supplementary Table S1.

### Protein Extraction and Western Blot

Cells were lyzed for whole protein extraction by RIPA lysis buffer containing protease inhibitors. Western blot analysis of the expression of collagen I, collagen II, aggrecan, and sox9 was carried out according to established protocols 17. GAPDH was used as the endogenous control. The relative amount of proteins was analyzed with a Quantity One software (Bio‐Rad) and normalized to GAPDH.

### Meniscectomy and MeSCs Injection

The anterior half of the bilateral medial meniscus was carefully resected (Supplementary Fig. S2), as described previously 14, 15. One and 2 weeks after surgery, intra‐articular injection of MeSCs‐7d‐in or MeSCs‐7d‐mix (mix of MeSCs‐7d‐in and 50% MeSCs‐7d‐out at 1:1 ratio) (1 × 10^6^ in 100 μl PBS) was performed on the left knee, while equal volume of PBS was injected into the right knee as negative control. Rats were sacrificed after 4 weeks (*n* = 5 per group) and 12 weeks (*n* = 5 per group) of treatment. For further details, see the Supplementary Materials. The study was conducted in accordance with NIH guidelines (NIH Pub No 85‐23, revised 1996), and the protocol was approved by the Ethics Committee of the Second Affiliated Hospital, School of Medicine, Zhejiang University, Hangzhou, China.

### Analysis of Regenerated Meniscus and OA

Fixed rat knee joints were decalcified with 10% EDTA until the samples became soft. H&E and S‐O staining of the regenerated meniscus were performed, as described previously 15. Macroscopically, regeneration of the injured meniscus was evaluated by area assay, and the degeneration of femoral and tibial articular cartilage was evaluated directly after ink staining 19, 20. Histological evaluation of the regenerated meniscus was performed using Ishida scoring 34. Moreover, Safranin O and fast green staining of cartilage were performed. Histological evaluation of OA was performed using modified Mankin's scoring 35. Four sections from each sample were graded blindly by three observers.

### Statistical Analysis

All quantitative data are presented as mean ± SD. At least three replicates for each experimental condition were performed, and the presented results are representative of these replicates. One‐way ANOVA and Fisher's predicted least‐square difference tests were performed to assess the statistical significance of results for multiple comparisons. Values of *p* < .05 were considered to be statistically significant.

## Results

### Morphological Evaluation of Rat Meniscus Tissue

#### *Haematoxylin and Eosin (H&E) Staining*

**Figure 1:**
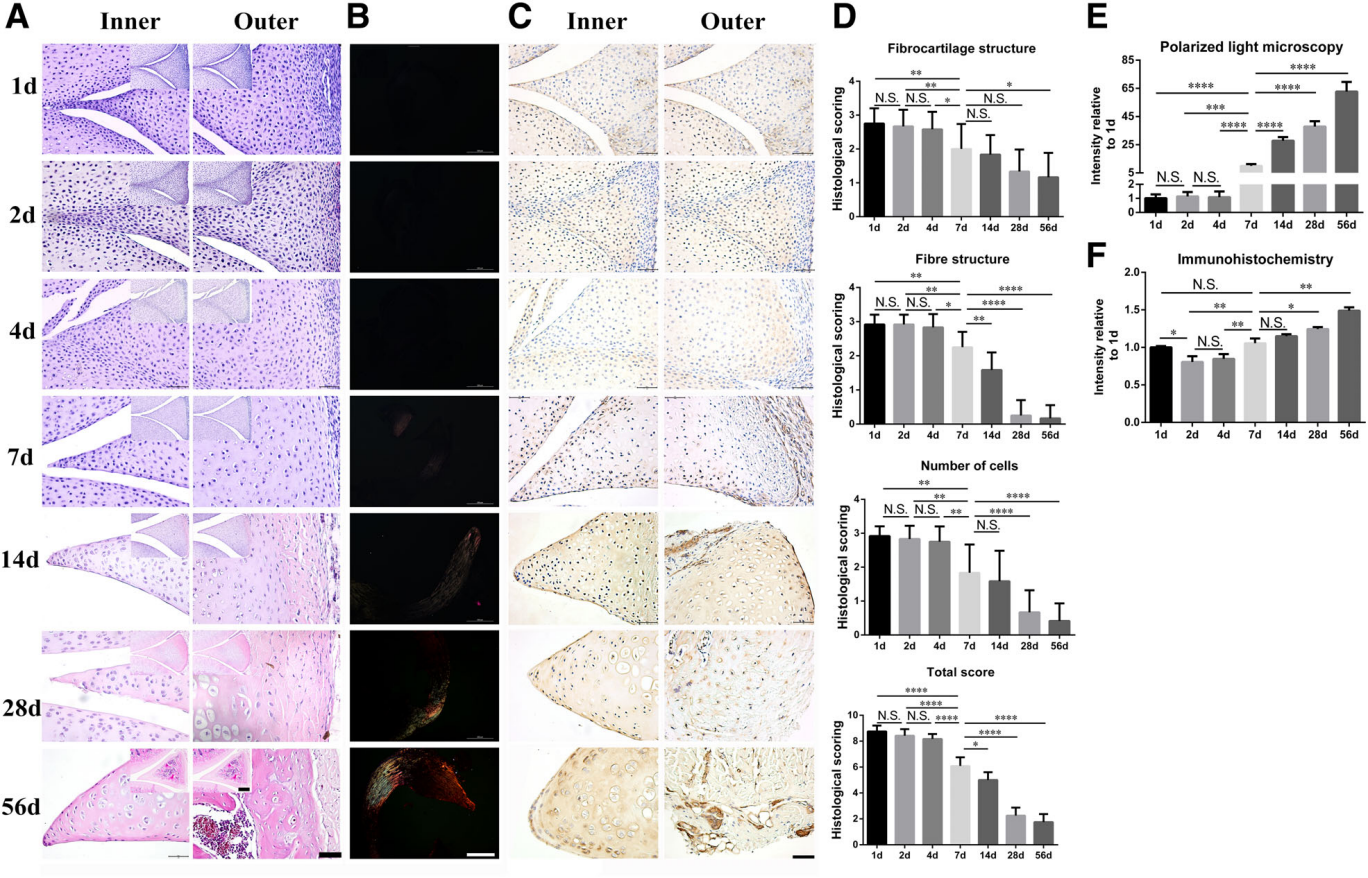
Histology score, polarized light microscopy, and immunohistochemistry evaluation of postnatal rat meniscus. **(A):** H&E staining on day 1, 2, 4, 7, 14, 28, and 56. **(B):** Polarized light microscopy images at day 1, 2, 4, 7, 14, 28, and 56. **(C):** Immunohistochemistry evaluation of collagen II expression on day 1, 2, 4, 7, 14, 28, and 56. **(D):** Histology score evaluation of fibrocartilage structure, fiber structure and number of cells. **(E):** Quantification of collagen fibers evaluation of polarized light microscopy images. **(F):** Quantification evaluation of collagen II of immunohistochemistry. Scale bars = 50 μm (A main panels, B and C), 200 μm (A insets 1d‐4d), 500 μm (A insets 7d‐56d). *Significant difference between two groups at *p* < .05. **Significant difference between two groups at *p* < .01. ***Significant difference between two groups at *p* < .001. ****Significant difference between two groups at *p* < .0001. N.S., no significant difference between two groups at *p* ≥ .05.

Meniscus of postnatal rats were harvested at day 1, 2, 4, 7, 14, 28, and 56. H&E staining of sagittal sections revealed that the meniscus experienced marked changes in tissue morphology, mainly in three parameters (Fig. 1A, 1D). First, tissue contains more cartilage structure in the inner region with time. Cells with obvious cartilage lacunae were found on day 7. Second, tissue contains more mature fibrous structures in the outer region with time. Cells with obvious fibroblast morphology were observed on day 7. Third, the number of cells decreased with development (Fig. 1A). The histology score evaluation demonstrated that day 7 had lower score as compared to day 1, 2, 4, and higher or similar score as compared to day 14, 28, and 56 in fibrocartilage structure, fiber structure, number of cells, and total score (Fig. 1D). These data suggest that in the rat meniscus tissue, day 7 may be the best representative time point for different development stages.

#### *Polarized Light Microscopy*

Histological transverse sections were observed under polarized light microscopy to evaluate the maturation level of collagen fibers (Fig. 1B, 1E). It was found that fibrous tissue could not be observed on day 1, 2, and 4, but was partially observed on day 7. Notably, golden‐yellow collagen fibers could be seen under polarized light microscopy with the day 56 meniscus tissue (Fig. 1B). Quantification of collagen fibers evaluation of polarized light microscopy images demonstrated that day 7 had higher intensity as compared to day 1, day 2, day 4 and lower intensity as compared to day 14, 28, and 56 (Fig. 1E). These data suggest that in the rat meniscus tissue, day 7 may be the best representative time point for different development stages.

#### *Immunohistochemistry Evaluation*

We evaluated the expression of collagen II in the meniscus of postnatal rats using immunohistochemistry (Fig. 1C, 1F). It was found that stronger staining for collagen II was observed on day 1, 7, and later, while weaker staining was observed on day 2 and 4 (Fig. 1C). Quantification evaluation of collagen II of immunohistochemistry found that day 1 and 7 had higher expression as compared to day 2 and 4 (Fig. 1F).

Together, these results suggest that the rat meniscus tissue exhibited marked changes in tissue morphology during development, with day 7 being the most representative time point of different developmental stages.

### Comparison of MeSCs at Different Developmental Stages and Tissue Regions

#### *Identification and Characterization of MeSCs at Different Developmental Stages*

**Figure 2:**
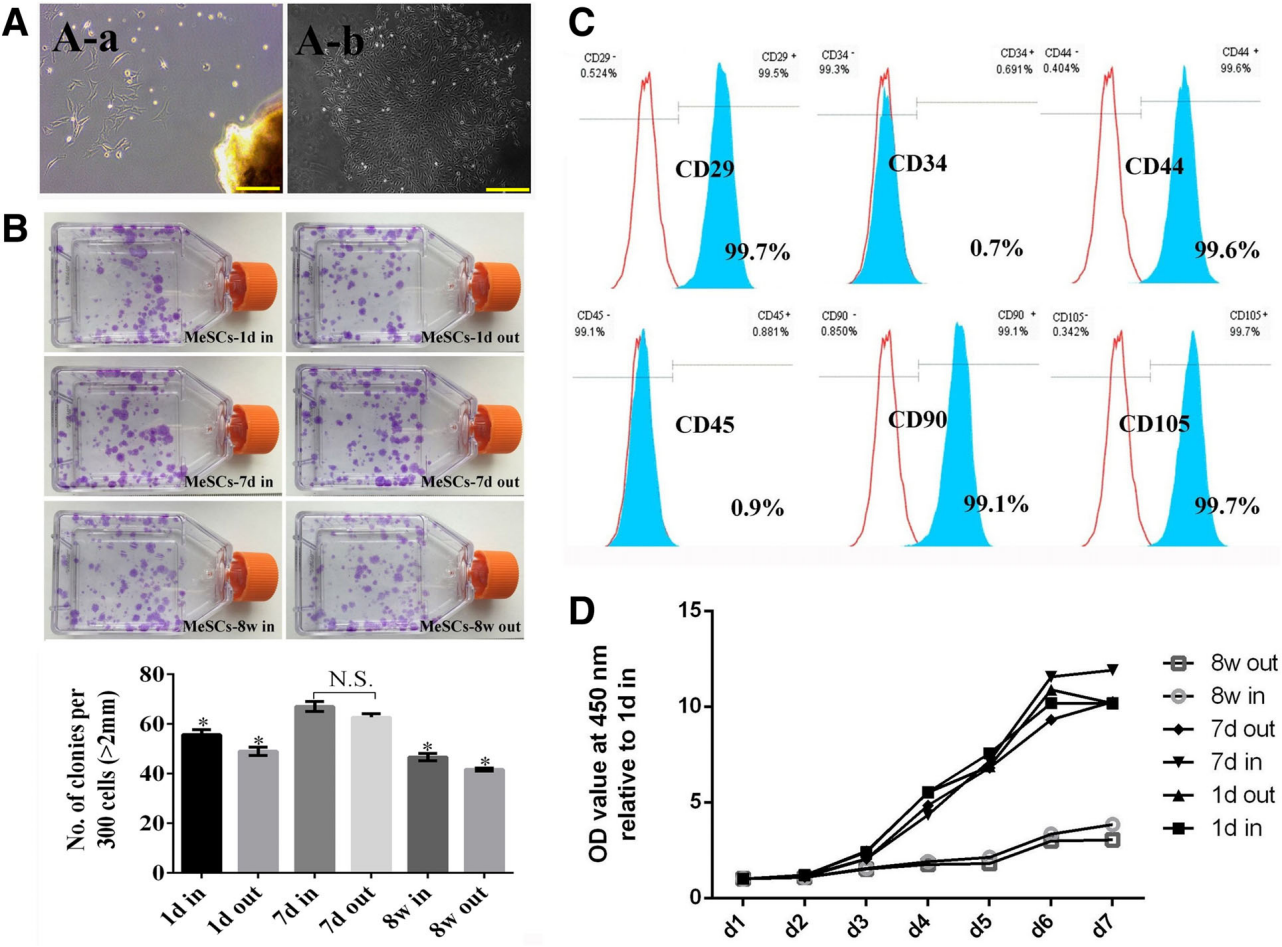
Characterization and comparison of MeSCs at different developmental stages and tissue regions. **(A‐a):** Cells adhered and spread from meniscus tissues. **(A‐b):** Colonies formed from a single cell. **(B):** Colony formation capacity comparison among different MeSCs subpopulations stained by crystal violet. **(C):** Comparison of MSCs surface marker expression by flow cytometry. **(D)**: Comparison of cell proliferation rates from 1 to 7 days in culture. Scale bars = 200 μm (A‐a), 500 μm (A‐b). *Significant difference relative to MeSCs‐7d‐in or MeSCs‐7d‐out at *p* < .05. N.S., no significant difference between two groups at *p* ≥ .05.

According to the histological results of the meniscus tissue at different developmental stages and tissue regions, we found that there were stark morphological differences between the inner and outer region of the meniscus, and that the observed changes in tissue morphology were mainly on day 1, day 7, and week 8. So, we further analyzed the differences in MeSCs isolated from these three representative time points and two regions. It was found that the large polygonal and star‐shaped cells adhered and spread from the meniscus tissues (Fig. 2A‐a). After low‐density culture, some single cells formed colonies in the dish. The colonies formed from MeSCs were heterogeneous in size, shape, and cell density, possibly reflecting differences in cell origin from the inner and outer regions of the meniscus. After several passages, a homogeneous population of MSC‐like cells was obtained (Fig. 2A‐b).

#### *Comparison of Colony Formation Capacity*

Methyl violet staining was used to compare the colony formation capacities of MeSCs subpopulations extracted from the inner or outer regions of the postnatal meniscus tissue at day 1, day 7, and week 8. All MeSCs possessed high capacities to form colonies, however, MeSCs‐7d‐in and MeSCs‐7d‐out had a significantly higher capacity to form colonies, as compared to the other four MeSCs subpopulations (Fig. 2B).

#### *Comparison of Surface Marker Expression*

The flow cytometry results showed that MeSCs showed similar expression profiles of CD markers as MSCs. They all expressed high levels of MSC markers, including CD29, CD44, CD90, and CD105, but were negative for the expression of hematopoietic markers such as CD34 and CD45(Fig. 2C and Supplementary Fig. S3).

#### *Comparison of Proliferative Rates*

The proliferation rates of MeSCs derived from day 1, day 7, and week 8 from the inner or outer regions of the meniscus tissue were compared using CCK‐8. Up to 3 days in culture, there were no significant differences between the MeSCs. From 4 days of culture onward, different OD values could be observed. At 7 days in culture, MeSCs‐7d‐in showed higher OD values as compared to the other five MeSCs subpopulations (Fig. 2D).

#### *Comparison of Differentiation Potential to Mesenchymal Lineages*

**Figure 3:**
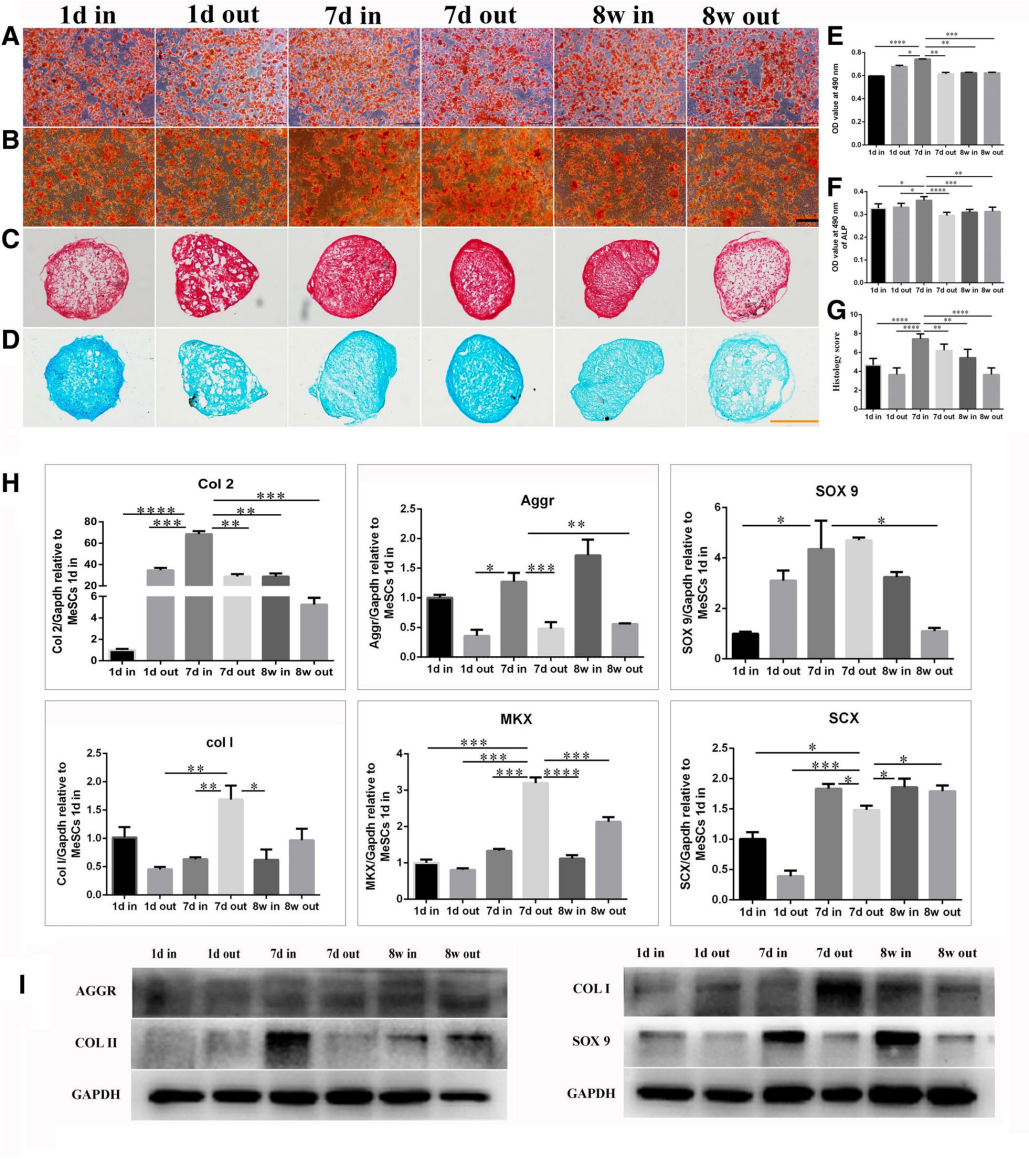
Comparison of mesenchymal lineage differentiation potential, the expression of genes, and proteins among MeSCs derived from different developmental stages and tissue regions. **(A, B):** Oil red staining for adipogenic differentiation after 14 days was performed and compared. **(C, D):** ARS staining for osteogenic differentiation after 14 days was performed and compared. **(E–G):** S‐O staining and alcian blue staining for chondrogenic differentiation after 28 days were performed and histology scores were compared. **(H):** The expression levels of genes Col2, Aggr, SOX9, Col1, Mkx, and Scx were compared by qPCR. **(I):** The expression levels of proteins Col2, Aggr, SOX9, and Col1 were compared by Western blot. Scale bars = 200 μm. *Significant difference between two groups at *p* < .05. **Significant difference between two groups at *p* < .01. ***Significant difference between two groups at *p* < .001. ****Significant difference between two groups at *p* < .0001.

The adipogenic differentiation assay showed that all MeSCs exhibited numerous lipid droplets; an indicator of adipogenesis which can be detected by Oil Red O staining (Fig. 3A). It was found that the adipogenic differentiation potential of MeSCs‐7d‐in was significantly higher than the other five MeSCs groups (Fig. 3E). The osteogenic differentiation assay showed that all MeSCs had the capacity to undergo osteogenic differentiation, as confirmed by the Alizarin Red S staining of mineralized calcium deposits (Fig. 3B). The ALP level in the MeSCs‐7d‐in group was significantly higher than the other five MeSCs groups (Fig. 3F). With regard to the chondrogenic differentiation assay, all MeSCs groups exhibited consolidated spherical tissue formation which was confirmed by S‐O staining (Fig. 3C) and alcian blue staining (Fig. 3D). The histology score evaluation demonstrated that MeSCs‐7d‐in had stronger chondrogenic differentiation potential as compared to the other five MeSCs groups (Fig. 3G).

#### *Comparison of Gene Expression*

It was found that MeSCs‐7d‐in expressed higher levels of Col II than the other five MeSCs groups. Much higher expression of Aggr was found in the MeSCs‐7d‐in group, as compared to MeSCs‐1d‐out, MeSCs‐7d‐out, and MeSCs‐8w‐out. Much higher expression of Sox9 was found in MeSCs‐7d‐in, as compared to MeSCs‐1d‐in and MeSCs‐8w‐out.

Much higher expression of Col I was found in MeSCs‐7d‐out, as compared to MeSCs‐1d‐out, MeSCs‐7d‐in, and MeSCs‐8w‐in. Meanwhile, it was found that MeSCs‐7d‐out expressed higher levels of Mkx than the other five MeSCs groups. Much higher expression of Scx was found in MeSCs‐7d‐out, as compared to MeSCs‐1d‐out and MeSCs‐1d‐in. However, lower expression of Scx was found in MeSCs‐7d‐out, as compared to MeSCs‐7d‐in, MeSCs‐8w‐out, and MeSCs‐8w‐in (Fig. 3H).

#### *Comparison of Protein Expression*

Western blotting was performed to quantify the level of specific protein expression in MeSCs. MeSCs‐7d‐in expressed significantly higher levels of Col II and Sox9, which are the most important indicators of chondrogenesis than others. Meanwhile, MeSCs‐7d‐out expressed significantly higher levels of Col I, which is one of the most important indicators of tenogenesis, as compared to other groups (Fig. 3I).

Together, these results suggest that MeSCs possess stem cell properties similar to MSCs. However, MeSCs‐7d‐in exhibited the highest self‐renewal capacity, cell proliferation, and differentiation potential toward mesenchymal lineages and the highest expression levels of chondrogenic genes and proteins, as compared to MeSCs derived from other regions and developmental stages of meniscus tissue.

### Effects of Intra‐Articular Injection of MeSCs on Meniscus Repair

**Figure 4:**
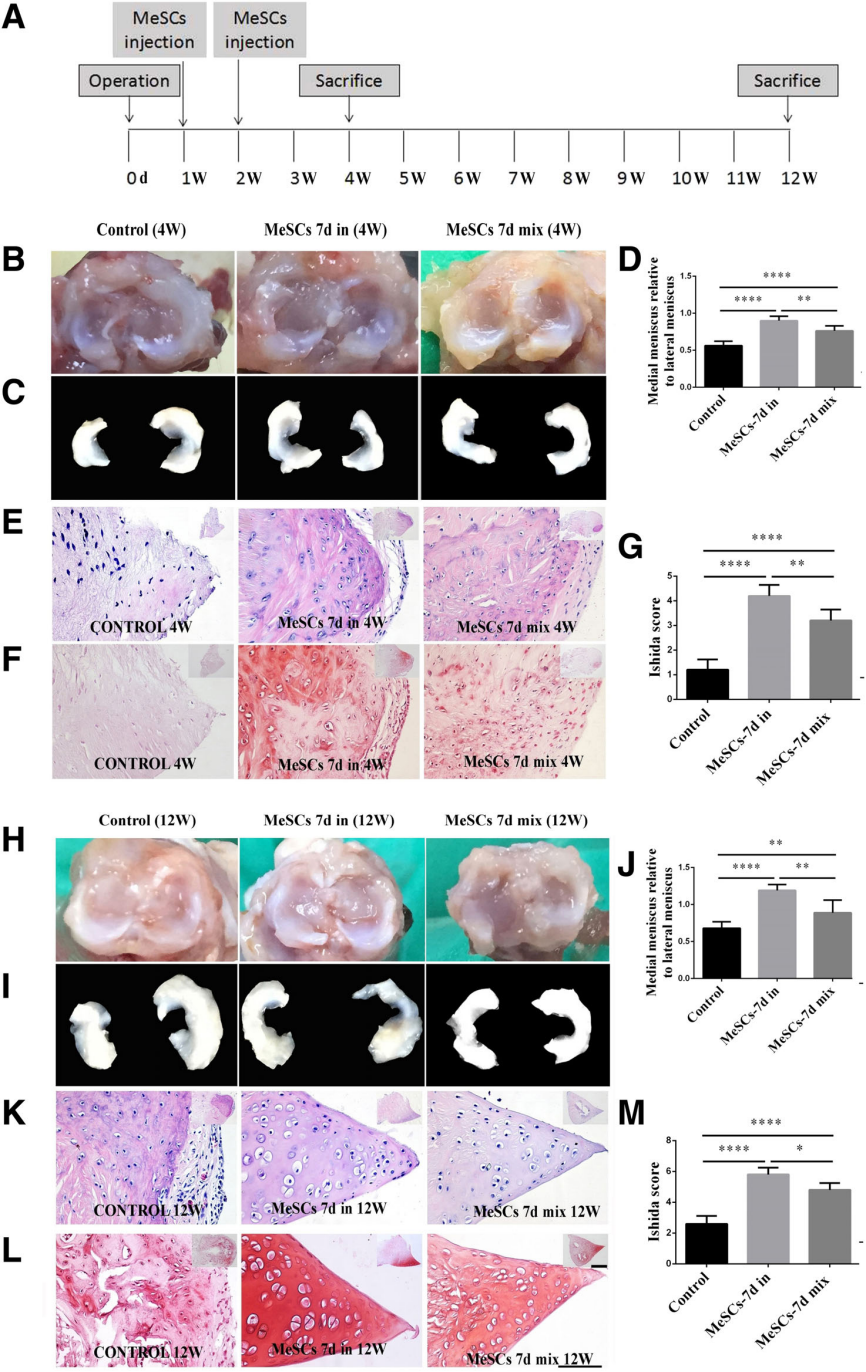
The result of meniscus repairs after intra‐articular injection of MeSCs. **(A):** Experimental design for the in vivo treatment of meniscus injury with MeSCs. **(B, C, D):** Gross morphology (B, C) and area assays (D) of the regenerated meniscus among the MeSC‐7d‐in treated group, MeSCs‐7d‐mix treated group, and control group at 4 weeks post‐implantation. **(E, F, G):** H&E staining (E), S‐O staining (F), and Ishida score (G) of regenerated meniscus among the MeSC‐7d‐in treated group, MeSCs‐7d‐mix treated group, and control group at 4 weeks post‐implantation. **(H, I, J):** Gross morphology (H, I) and area assays (J) of the regenerated meniscus among the MeSC‐7d‐in treated group, MeSCs‐7d‐mix treated group, and control group at 12 weeks post‐implantation. **(K, L, M):** H&E staining (K), S‐O staining (L), and Ishida score (M) of regenerated meniscus among the MeSC‐7d‐in treated group, MeSCs‐7d‐mix treated group, and control group at 12 weeks post‐implantation. Scale bars = 50 μm (main panels), 200 μm (insets). *Significant difference between two groups at *p* < .05. **Significant difference between two groups at *p* < .01. ****Significant difference between two groups at *p* < .0001. Abbreviations: MeSCs, meniscus derived stem cells; W, weeks.

According to the results of comparison of MeSCs at different developmental stages in vitro, it was found that MeSCs‐7d‐in might be ideal seed cells. At the same time, it was found that the inner and outer MeSCs at this time point had their own characteristics. Moreover, there are stark morphological differences between the inner and outer regions of the meniscus. So, we further analyzed the feasibility and effectiveness of MeSCs regeneration at this time point to repair meniscus tissue, and compare the effects between intra‐articular injection of MeSCs‐7d‐in and MeSCs‐7d‐mix in vivo (Fig. 4A).

At 4 and 12 weeks postmeniscectomy, the gross morphology showed that MeSCs‐7d‐in and MeSCs‐7d‐mix injected groups exhibited more neo‐tissue formation within the anterior part of the meniscus defect, as compared to the control group. Furthermore, MeSCs‐7d‐in injection group exhibited more neo‐tissue formation compared to the MeSCs‐7d‐mix group in terms of neo‐tissue formation (Fig. 4B–4D, 4H–4J). Histologically, the H&E and S‐O staining of meniscus injected with both MeSCs‐7d‐in and MeSCs‐7d‐mix exhibited more neo‐tissue formation and better‐defined shapes, had higher deposition levels of cartilage matrix similar to normal meniscus, as well as have higher histological scores compared to the control group. Furthermore, the histological score for meniscus repair was higher in the MeSCs‐7d‐in injection group than in the MeSCs‐7d‐mix injection group (Fig. 4E–4G, 4K–4M).

### Effects of Intra‐Articular Injection of MeSCs on the supprEssion of the Progression of OA

**Figure 5:**
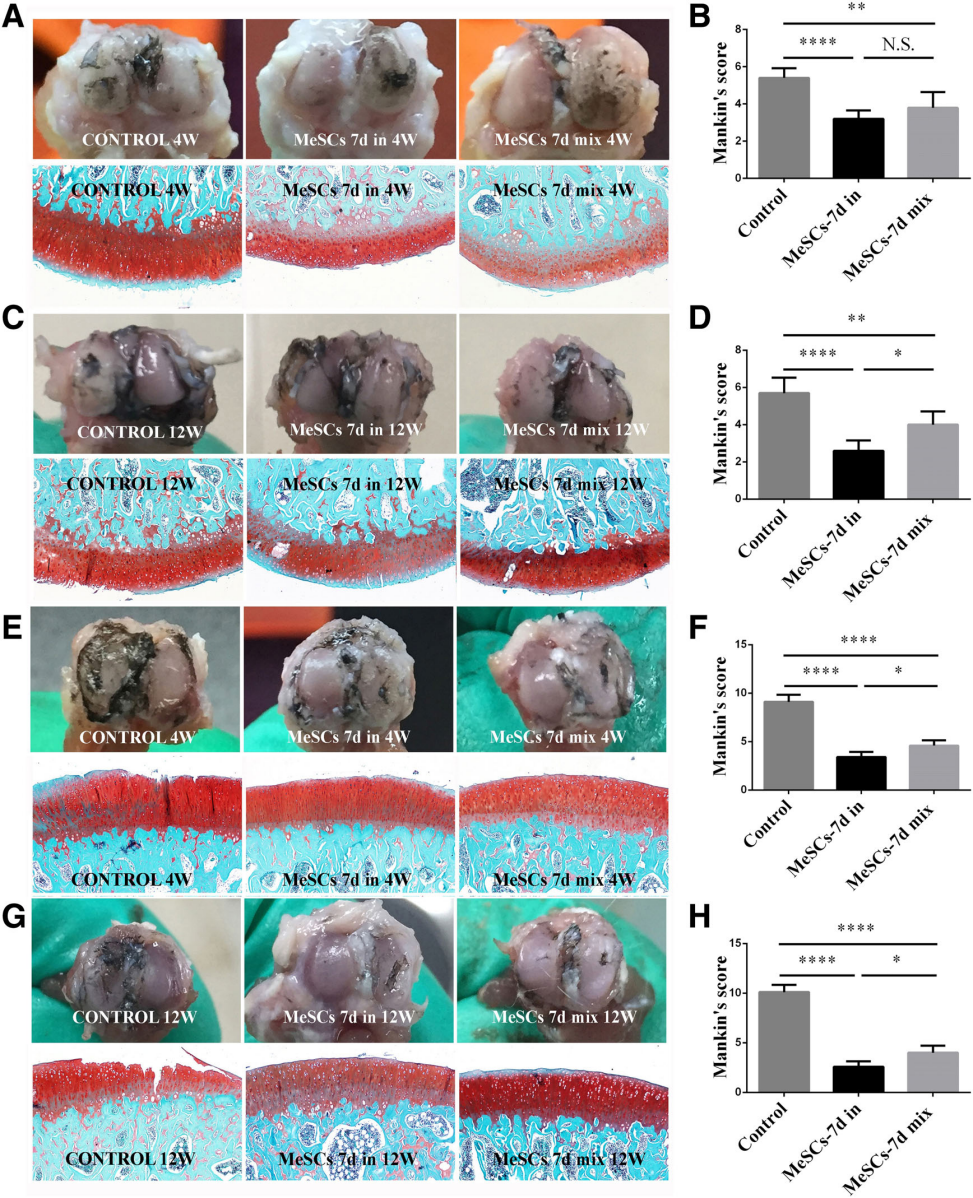
Suppression of experimental osteoarthritis after intra‐articular injection of MeSCs. **(A, B):** Gross morphology upon staining with Indian ink, S‐O staining and Mankin's score of the medial femur cartilage surface among the MeSC‐7d‐in treated group, MeSCs‐7d‐mix treated group, and control group at 4 weeks postimplantation. **(C, D):** Gross morphology upon staining with Indian ink, S‐O staining and Mankin's score of the medial tibia cartilage surface among the MeSC‐7d‐in treated group, MeSCs‐7d‐mix treated group, and control group at 12 weeks post‐implantation. **(E, F):** Gross morphology upon staining with Indian ink, S‐O staining and Mankin's score of the medial femur cartilage surface among the MeSC‐7d‐in treated group, MeSCs‐7d‐mix treated group, and control group at 12 weeks post‐implantation. **(G, H):** Gross morphology upon staining with Indian ink, S‐O staining and Mankin's score of the medial tibia cartilage surface among the MeSC‐7d‐in treated group, MeSCs‐7d‐mix treated group, and control group at 12 weeks post‐implantation. Scale bars = 200 μm. *Significant difference between two groups at *p* < .05. **Significant difference between two groups at *p* < .01. ****Significant difference between two groups at *p* < .0001. Abbreviations: MeSCs, meniscus‐derived stem cells; W, weeks.

At 4 and 12 weeks postmeniscectomy, the gross morphology showed that craters were present on the articular cartilage surface of the medial femoral condyle and particularly in the medial tibial plateau of the control, while both the MeSCs‐7d‐in and MeSCs‐7d‐mix treated groups were less affected. Microscopically, cartilage degeneration was observed at both the surface of the medial femoral condyle and particularly in the medial tibial cartilage in the control groups. However, less damage of the cartilage could be observed in both the MeSCs‐7d‐in and MeSCs‐7d‐mix treated groups (Fig. 5A, 5C, 5E, 5G).

Mankin's score of both the MeSCs‐7d‐in injection group and the MeSCs‐7d‐mix injection group at the surface of the medial femoral condyle and the medial tibial cartilage were higher as compared to the control group. Furthermore, Mankin's score were higher in the MeSCs‐7d‐in injection group than in the MeSCs‐7d‐mix injection group at the surface of the medial femoral condyle 12 weeks postmeniscectomy and medial tibial cartilage both 4 and 12 weeks postmeniscectomy. However, there were no significant differences between the MeSCs‐7d‐in and MeSCs‐7d‐mix injection groups at the surface of the medial femoral condyle 4 weeks postmeniscectomy (Fig. 5B, 5D, 5F, 5H).

## Discussion

Our study (a) performed the first evaluation of tissue structure and morphological changes occurring in different regions during development of the postnatal rat meniscus; (2) successfully isolated, identified, and compared MeSCs from the inner or outer regions of meniscus at three different development stages. MeSCs‐7d‐in exhibited the highest self‐renewal capacity, cell proliferation, and differentiation potential toward mesenchymal lineages in vitro; and (c) demonstrated intra‐articular injection of allogenous MeSCs promoted the regeneration of meniscus and protected the joint surface in an experimental OA model (rat knee joint) in vivo. Collectively, these results indicated that MeSCs‐7d‐in may be a promising cell source for meniscus regeneration and OA prevention in the future.

### MeSCs Can be Ideal Seed Cells for Meniscus Regeneration and Reducing the Progression of OA

Our team first proposed the concept of MeSCs which possess self‐renewal capacity, multilineage differentiation potential and have immunosuppressive properties 14, 15. Ding et al. found that MSeCs have universal stem cell characteristics including clonogenicity, multipotency, and self‐renewal capacity, together with a propensity for chondrogenic differentiation 17. Gui et al. found that a high level of collagen II expression was detected in meniscus‐derived stem cells. Moreover, these cells appeared to have a pronounced tendency to differentiate into the chondrogenic lineage 18. Our previous studies have used MeSCs for meniscus tissue engineering, which has shown much promise for meniscus regeneration, thereby delaying or reducing the progression of OA induced by meniscectomy 14, 15. These data thus suggest that MeSCs have great potential for the regeneration of meniscus. In our in vitro study, we found that all populations expressed a high level of MSC markers but lacked expression of hematopoietic markers, which reveals that MeSCs still remain undifferentiated and preserved their self‐renewal capacity (Fig. 2C). The in vivo study demonstrated that the MeSC‐injection group exhibited more neo‐tissue formation and better histological staining results than the control group (Figs. 4, 5), thus confirming the key role of MeSCs in the process of meniscus healing. Our findings thus confirmed articular cartilage protection through meniscus regeneration induced by intra‐articular injection of MeSCs in rats undergoing meniscectomy.

There are some studies concerning the difference between young meniscus cells versus aged meniscus cells or young meniscus tissues versus aged meniscus tissues 22, 29. However, to date, no report has yet mentioned the difference between young meniscus cells and aged meniscus cells for regeneration of injured meniscus. Therefore, it is necessary to evaluate MeSCs in a more accurate developmental model, and to identify more suitable subpopulations of MeSCs to achieve better tissue engineering regeneration.

### MeSCs‐7d Is a Better Cell Source than MeSCs‐1d or MeSCs‐8w In Vitro

The ideal cells for meniscus regeneration should express more chondrogenic and angiogenic genes and proteins 36. Several studies have reported the architectural differences between young meniscus tissues and aged meniscus tissues in terms of tissue composition and organization 20, 21, 22, 23, 24, 37, 38. Hyde et al. found that meniscus cells started to express collagen II at 1 week after birth, and the expression levels of collagen II and angiogenesis in meniscus were significantly higher in the neonatal meniscus than in young and adult menisci 29. Pazin et al. showed that the gene expression of growth factors declined with age, and several meniscus‐enriched genes were expressed either in the inner or outer meniscus 30. Furthermore, Zhou et al. compared tendon stem/progenitor cells (TSPCs) derived from young rats (3–4 month) and old rats (24–26 month), and found that young TSPCs had stronger proliferation and colony formation capacities than older cells 39. Zhou et al. compared young human BMSCs with old human BMSCs, and found that there is an age‐dependent decrease in proliferation and osteoblast differentiation 25. Zhao et al. compared nucleus pulposus MSCs (NPMSCs) isolated from young (3‐month‐old) and old (14‐month‐old) rats, and found that young NPMSCs presented better proliferation, colony‐forming, and multilineage differentiation capacities than old NPMSCs 26. All of these studies indicate that the function and fate of MSCs are age dependent during tissue development, with younger MSCs having better regeneration potential compared to old MSCs. Nevertheless, Chen et al. compared TSPCs derived from 1‐day postnatal rat to TSPCs derived from 7 days and 56 days postnatal rats. They found that TSPCs derived from day 7 Achilles tendon tissue is a superior cell source as compared to TSPCs derived from 1 day and 56 days postnatal tissue 27. Hence, it is suggested that MSCs at younger developmental stages might not necessarily be better.

Therefore, we compared the differences in the tissue and MeSCs of postnatal rat meniscus at different developmental stages in our study. Striking changes in tissue structure and morphology were observed over time via H&E staining, polarized light, and immunohistochemistry evaluation (Fig. 1). We chose 7 days as an important time point to study the MeSCs based on the finding that obvious cartilage lacunae within the inner region and striking fibroblast‐like cells within the outer region were found around this time point, as were seen with H&E staining. The 7 day time point was also chosen because fibrous structures in the outer region appear. Third, the number of cells decreased significantly on day 7 (Fig. 1A, 1D). This finding was further confirmed under polarized light microscopy observation, in which the meniscus tissue structure could be observed in 7 day postnatal tissue, which was not the case with the 1 day postnatal tissue (Fig. 1B, 1E). We evaluated the expression of collagen II in the meniscus of postnatal rats via immunohistochemistry. It was found that stronger staining for collagen II was observed on day 1, day 7, and later, while weaker staining was observed on day 2 and day 4 (Fig. 1C, 1F). These data were similar to previous results 28.

MeSCs‐7d has a significantly higher colony formation capacity, cell proliferation, and chondrogenic differentiation potential, as well as higher chondrogenic (7d‐in) or tenogenic (7d‐out) gene and protein expression levels as compared to both MeSCs‐1d and MeSCs‐8w, indicating that they possess intrinsically better self‐regenerative stem cell properties (Figs. 2, 3). In further studies, molecular network and microenvironment of MeSCs will be examined. Taken together, MeSCs‐7d can be a promising cell source for meniscus regeneration.

Meniscus is a heterogeneous tissue. There are some studies concerning the difference between inner meniscus cells and outer meniscus cells or inner meniscus tissues and outer meniscus tissues 40, 41. Mary et al. found that BMSCs cocultured with primary meniscal fibrochondrocytes produced significantly more glycosaminoglycan (GAG) and aggregate modulus 42. A mixture of MeSCs‐7d‐in and MeSCs‐7d‐out was hypothesized to facilitate meniscus regeneration and articular cartilage protection. Nevertheless, to date, no report has yet mentioned the difference between using mixed MeSCs and MeSCs from the inner region for regeneration of injured meniscus. Therefore, it is meaningful to evaluate MeSCs from specific regions to identify more suitable MeSCs subpopulations for achieving better tissue engineering regeneration. Hence, we selected a mixture of MeSC‐7d‐in and MeSCs‐7d as seed cells for our further in vivo study.

### MeSCs‐7d‐in Is a Promising Cell Source for Meniscus Regeneration and Articular Cartilage Protection In Vivo

Previous studies confirmed that chondrocyte‐like cells are surrounded mainly by collagen II fibers in the inner region of the menisci, while, fibroblast‐like cells reside in the outer region surrounded mainly by collagen I fibers 24, 43. Additionally, there is partial vascularization in the outer region, whereas the inner region does not contain blood vessels 29, 43. Giancamillo et al. found that expression of the angiogenic factor were significantly increased from the inner to the outer part of the meniscus 29. Our results also showed that there are great morphological differences between the inner and outer regions of the meniscus.

Upton et al. found that the innermost regions of the meniscus constitutively expressed higher mRNA levels of proteins that are highly expressed in articular cartilage. In contrast, the outer meniscus was found to contain higher gene expression for proteins associated with fibrous tissues 44. Fuller et al. found that the inner meniscus was more cartilaginous containing more GAG and expressing more aggrecan and type II collagen than the outer zones 40. Furthermore, some researches were able to engineer a meniscus, which is cartilage‐like cells with primarily type II collagen at the inner portion and fibrocartilage‐like cells with primarily type I collagen at the outer portion for total meniscus replacement 45, 46. MeSCs are the only stem cells that have been found in meniscus tissue 15. Therefore, there might be a difference between inner MeSCs and outer MeSCs. Our in vitro results showed that MeSCs from the inner and outer regions have a significantly higher capacity for chondrogenic and tenogenic gene and protein expression, respectively (Fig. 3H, 3I).

In previous studies, our team have reported that intra‐articular injection of MeSCs enhances the regeneration of the injured meniscus and delay or reduce the progression of OA induced by meniscectomy 14, 15. Huang et al 47 also reported that MeSCs may play a greater role in repair or regeneration of meniscus. Currently, injection of MeSCs has not been used clinically. However, injection of other MSCs has been utilized in many fields 48, 49, 50, 51, 52, 53, 54. Hence, it is necessary and meaningful to explore more suitable MeSCs for clinical tissue engineering regeneration in the future. Thus, we selected MeSC‐7d‐in and MeSCs‐7d‐mix as seed cells for our further in vivo investigation. Our studies demonstrate that both the injected MeSCs‐7d‐in and MeSCs‐7d‐mix play an important role in modulating meniscus regeneration and cartilage protection (Figs. 4, 5). These results were consistent with previous studies 14, 15, 47. Furthermore, MeSCs‐7d‐in the injection group enhanced more neo‐tissue formation and suppressed OA progression better, as compared to the MeSCs‐7d‐mix injection group (Figs. 4, 5). It is assumed that the inner region contains mainly chondrocytes and the outer region contains mainly fibroblasts. Mixed MeSCs injection might achieve better meniscus regeneration and articular cartilage protection than the inner MeSCs alone. Nevertheless, our results suggest that MeSCs‐7d‐mix injection group is not as effective as inner MeSCs alone (MeSCs‐7d‐in injection group). The mechanism of this phenomenon may be similar to OA progression 55, 56, 57. Although chondrocytes may be transformed into fibroblasts in the process of regeneration, fibroblasts cannot differentiate into chondrocytes in reverse. Hence, fibroblasts from MeSCs‐7d‐out may be a competitive inhibitor of chondrocytes from MeSCs‐7d‐in in the regeneration of meniscus and protection of articular cartilage.

A few limitations of our study still exist. First, although our results found that rat MeSCs derived from 7 day is better than MeSCs derived from 1 day or 8 week, it is necessary to optimize and explore the optimal MeSCs more accurately in a further study. Second, human meniscus is different from rat meniscus in a number of major structural features. So, characterization and comparison of human MeSCs at different stages and tissue regions are needed for further studies. Hence, in our future study, we will optimize MeSCs to improve the efficacy of intra‐articular injection of MeSCs in human meniscus repair.

## Conclusion

In summary, both our in vitro and in vivo studies demonstrated that there are significant differences in meniscus tissues and MeSCs in different tissue regions and at different developmental stages. MeSCs‐7d‐in was found to have better cell proliferation, colony formation capacity, mesenchymal‐lineage differentiation potential, chondrogenic gene, and protein expression as compared to other MeSCs subpopulation. Intra‐articular injection of MeSCs‐7d‐in promoted meniscus regeneration and protected the joint surface cartilage.

## Author Contributions

S.H.: conception and design, collection of data, data analysis and interpretation, manuscript writing; D.R., J.R., X.C., Z.Y., C.T., J.C., and J.H.: collection and assembly of data, data analysis and interpretation, manuscript writing; Y.C.: collection and assembly of data, administrative support, data analysis and interpretation; B.H.: revision of manuscript; W.S.: conception and design, provision of study material, data analysis and interpretation, final approval of manuscript; W.C.: conception and design, data analysis and interpretation, final approval of manuscript; H.O.: final approval of manuscript.

## Disclosure of Potential Conflicts of Interest

The authors declared no potential conflicts of interest.

## Supporting information


**Supplementary Figure 1. Schematic representation of the inner and outer regions of the meniscus**. The midline between the innermost and outermost sides of the meniscus were used as the boundary of inner and outer regions.

**Supplementary Figure 2. Schematic representation of the experimental design for the OA model**. (A): Schematic representation of the experimental design for the OA model. (B): Intraoperative view of the experimental design for the OA model. The anterior half of the medial meniscus was resected with the medial collateral ligament as the boundary. The medial collateral ligament (triangle) and the anterior half of the medial meniscus (black arrows).

**Supplementary Figure 3**. Comparison of MSCs surface marker expression of MeSCs‐1d‐in, MeSCs‐1d‐out, MeSCs‐7d‐out, MeSCs‐8w‐in, and MeSCs‐8w‐out by flow cytometry.

**Supplementary Figure 4. The expression levels of genes PPAR γ, Osteocalcin, and Runx2 were compared by qPCR**. *Significant difference between two groups at p< 0.05. **Significant difference between two groups at p< 0.01. N.S.No significant difference between two groups at p ≥ 0.05.

**Supplementary Table 1**. List of primer sequences used for real‐time polymerase chain reaction.

**Supplementary Table 2**. Meniscus histological histologic scoring system

**Supplementary Table 3**. The Animals in Research: Reporting in vivo Experiments (ARRIVE) Guidelines.

**Appendix** S1: Supplementary information

## Data Availability

The data that support the findings of this study are available from the corresponding author upon reasonable request.
