# Supplementary figures and images for: Characterization and Comparison of Postnatal Rat Meniscus Stem Cells at Different Developmental Stages
Source: Stem Cells Transl Med. 2019 Oct 22;8(12):1318–29. doi: 10.1002/sctm.19-0125 (PMC6877772; doi:10.1002/sctm.19-0125)

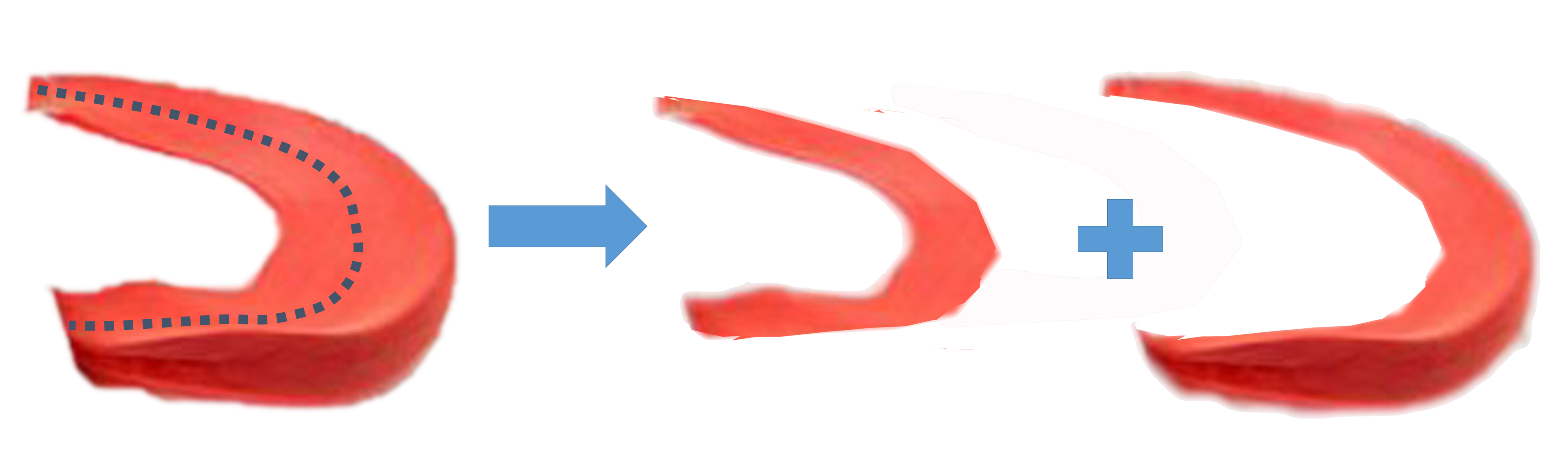

Supplement: Supplementary file 1 — Supplementary Figure 1. Schematic representation of the inner and outer regions of the meniscus. The midline between the innermost and outermost sides of the meniscus were used as the boundary of inner and outer regions. [file SCT3-8-1318-s001.tif]

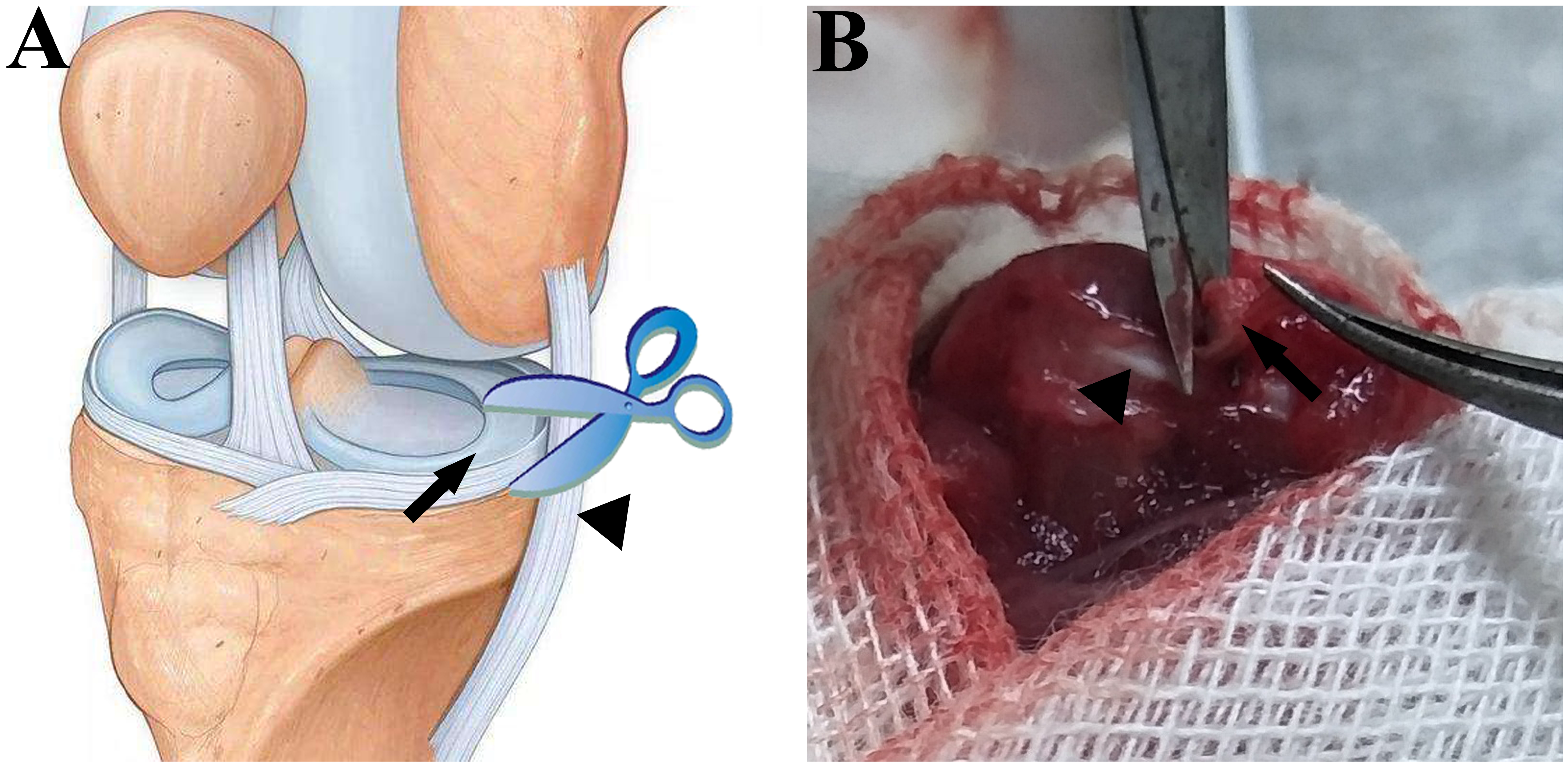

Supplement: Supplementary file 2 — Supplementary Figure 2. Schematic representation of the experimental design for the OA model. (A): Schematic representation of the experimental design for the OA model. (B): Intraoperative view of the experimental design for the OA model. The anterior half of the medial meniscus was resected with the medial collateral ligament as the boundary. The medial collateral ligament (triangle) and the anterior half of the medial meniscus (black arrows). [file SCT3-8-1318-s002.tif]

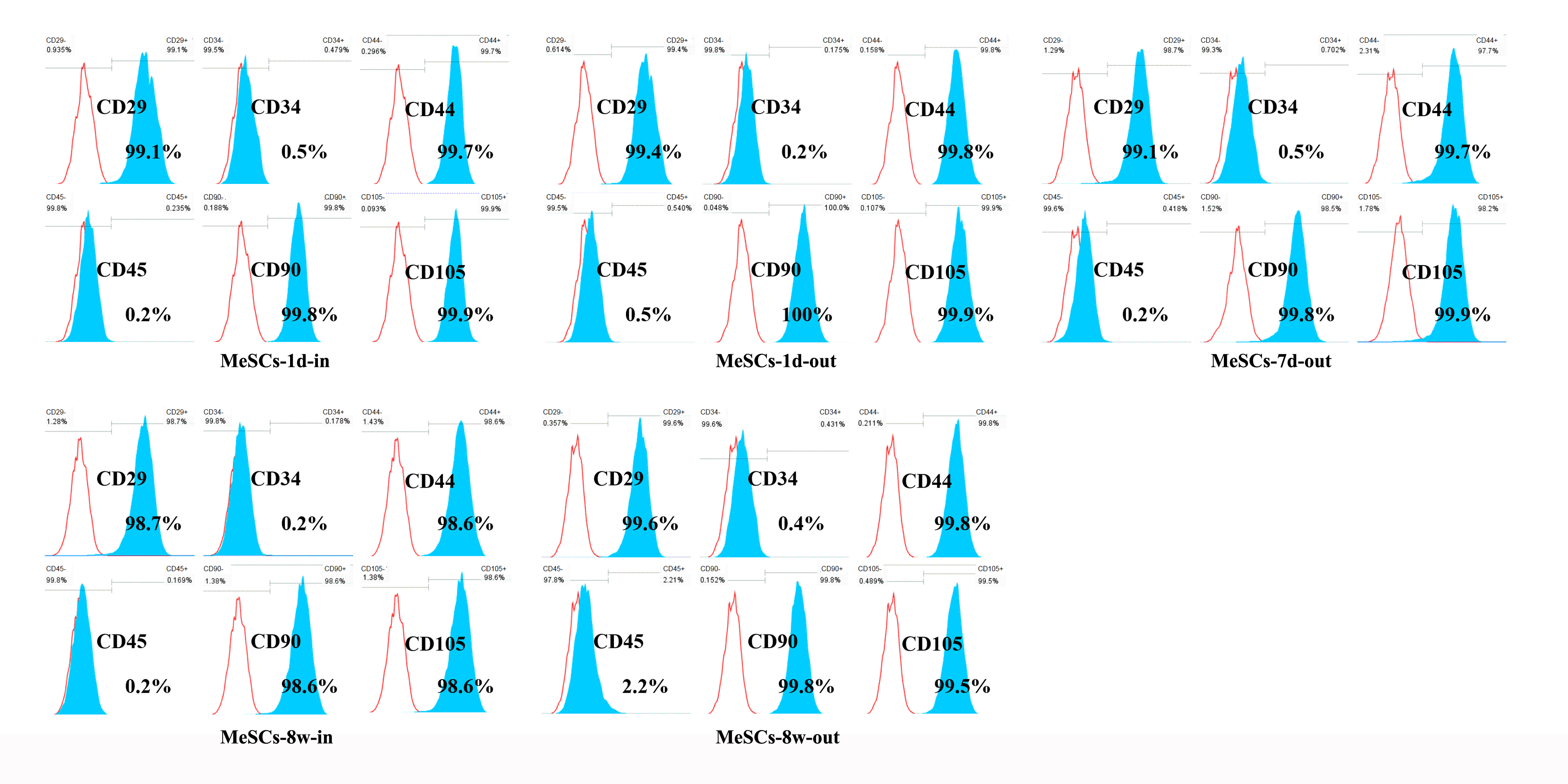

Supplement: Supplementary file 3 — Supplementary Figure 3. Comparison of MSCs surface marker expression of MeSCs‐1d‐in, MeSCs‐1d‐out, MeSCs‐7d‐out, MeSCs‐8w‐in, and MeSCs‐8w‐out by flow cytometry. [file SCT3-8-1318-s003.tif]

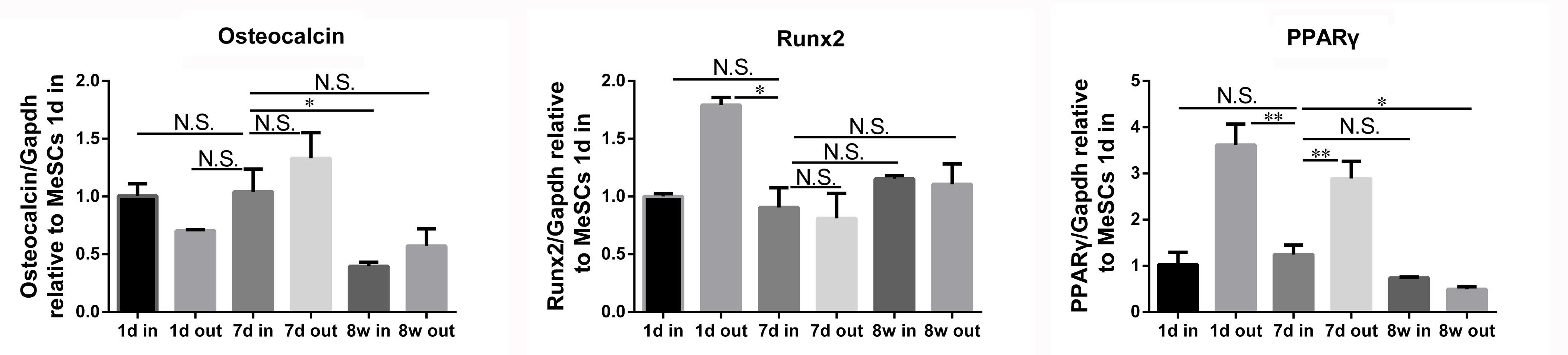

Supplement: Supplementary file 4 — Supplementary Figure 4. The expression levels of genes PPAR γ, Osteocalcin, and Runx2 were compared by qPCR. *Significant difference between two groups at p< 0.05. **Significant difference between two groups at p< 0.01. N.S.No significant difference between two groups at p ≥ 0.05. [file SCT3-8-1318-s004.tif]
