# Supplementary material for: Characterization and Comparison of Postnatal Rat Meniscus Stem Cells at Different Developmental Stages
Source: Stem Cells Transl Med. 2019 Oct 22;8(12):1318–29. doi: 10.1002/sctm.19-0125 (PMC6877772; doi:10.1002/sctm.19-0125)
Supplement: Supplementary file 5 — Supplementary Table 1. List of primer sequences used for real‐time polymerase chain reaction. [file SCT3-8-1318-s005.docx]

**Supplementary Table 1. List of primer sequences used for real-time polymerase chain reaction.**

| Genes | 5’-3’ | Primers |
| --- | --- | --- |
| Rat Collagen II | Forward | GGCCAGGATGCCCGAAAATTA |
|  | Reverse | CCCTCTCTCCCTTGTCACCAC |
| Rat SOX 9 | Forward | AAGTCGGTGAAGAATGGGCA |
|  | Reverse | GTCGGTGGACCCTGAGATTG |
| Rat Aggrecan | Forward | CTGGGTGGATGCAGAGAGAC |
|  | Reverse | TTGGTTTGGACGCCACTTCT |
| Rat Collagen I | Forward | TGGATGGCTGCACGAGT |
|  | Reverse | TTGGGATGGAGGGAGTTTA |
| Rat MKX | Forward | GAAGGTGAGGCACAAGCGACAG |
|  | Reverse | TCTGCGAGCCTAGAGCCAAGAG |
| Rat SCX | Forward | ACTACCACTGGCCAGAGACG |
|  | Reverse | ATCGCCGTCTTTCTGTCACG |
| Rat PPARγ | Forward | AGAGCCTTCAAACTCCCTCA |
|  | Reverse | GAGACATCCCCACAGCAAG |
| Rat Osteocalcin | Forward | GACCCTCTCTCTGCTCACTCT |
|  | Reverse | GACCTTACTGCCCTCCTGCTTG |
| Rat Runx2 | Forward | CCAACTTCCTGTGCTCCGTG |
|  | Reverse | GTGAAACTCTTGCCTCGTCCG |
| Rat GAPDH | Forward | GCAAGTTCAACGGCACAG |
|  | Reverse | CGCCAGTAGACTCCACGAC |

Abbreviations: GAPDH, glyceraldehyde-3-phosphate dehydrogenase
