# Supplementary material for: Characterization and Comparison of Postnatal Rat Meniscus Stem Cells at Different Developmental Stages
Source: Stem Cells Transl Med. 2019 Oct 22;8(12):1318–29. doi: 10.1002/sctm.19-0125 (PMC6877772; doi:10.1002/sctm.19-0125)
Supplement: Supplementary file 6 — Supplementary Table 2. Meniscus histological histologic scoring system [file SCT3-8-1318-s006.doc]

**Supplementary Table 2. Meniscus histological histologic scoring system**

| Points | Cell number density  (Cells /mm^2^) | Fibrocartilage structure  (Cartilage lacunae rate) | Fiber structure |
| --- | --- | --- | --- |
| 0 | Less than 1000 | Less than 25% | Well organized |
| 1 | 1000 to 2000 | 25% to 50% | General organized |
| 2 | 2001 to 3000 | 26% to 75% | Poor organized |
| 3 | More than 3000 | More than 75% | Disorganized |
