# Supplementary material for: Characterization and Comparison of Postnatal Rat Meniscus Stem Cells at Different Developmental Stages
Source: Stem Cells Transl Med. 2019 Oct 22;8(12):1318–29. doi: 10.1002/sctm.19-0125 (PMC6877772; doi:10.1002/sctm.19-0125)
Supplement: Supplementary file 7 — Supplementary Table 3. The Animals in Research: Reporting in vivo Experiments (ARRIVE) Guidelines. [file SCT3-8-1318-s007.docx]

**Supplementary Table 3. The Animals in Research: Reporting In Vivo Experiments (ARRIVE) Guidelines.**

| Item/  subitem | ARRIVE items and sub-items | Descriptor |
| --- | --- | --- |
| **1** | **Title** | Page 2. Title |
| **2** | **Abstract** | Page 5. Abstract |
|  | Introduction | Page 5 to 7. Introduction |
| **3** | **Background** | Page 5 to 7. Background |
| 3a | Motivation for and context of study | Page 5 to 7. Motivation for and context of study |
| 3b | Animal species and models justified | Page 7. Animal species and models justified |
| **4** | **Objectives** | Page 7. Objectives |
|  | Methods | Page 7, 8 and 11. Methods |
| **5** | **Ethical Statement** | Page 11. Ethical Statement |
| **6** | **Study design** | Page 11, 53 and 54. Study design |
| 6a | Number of groups | Page 11, 53 and 54. Number of groups |
| 6b | Randomization | Page 54. Randomization |
| 6c | Blinding | Page 53 and 54. Blinding |
| 6d | Experimental unit | Page 11, 53 and 54. Experimental unit |
| **7** | **Experimental procedures** | Page 11, 53 and 54. Experimental procedures |
| 7a | How | Page 11, 53 and 54. How |
| 7b | When | Page 11, 53 and 54. When |
| 7c | Where | Page 11, 53 and 54. Where |
| 7d | Why | Page 11, 53 and 54. Why |
| 7e | Drugs used | Page 53 and 54. Drugs used |
| **8** | **Experimental animals** | Page 53. Experimental animals |
| 8a | Species | Page 53. Species |
| 8b | Strain | Page 53. Strain |
| 8c | Sex | Page 53. Sex |
| 8d | Developmental stage | Page 53. Developmental stage |
| 8e | Weight | Page 53. Weight |
| 8f | Source | Page 53. Source |
| 8g | Health/immune status | Page 53 and 54. Health/immune status |
| **9** | **Housing and husbandry** | Page 53 and 54. Housing and husbandry |
| 9a | Type of cage/housing | Page 53. Type of cage/housing |
| 9b | Bedding material | Page 53. Bedding material |
| 9c | Type of facility | Page 53. Type of facility |
| 9d | Number of cage companions | Page 53. Number of cage companions |
| 9e | Light/dark cycle | Page 53. Light/dark cycle |
| 9f | Temperature | Page 53. Temperature |
| 9g | Type of food | Page 53. Type of food |
| 9h | Water access | Page 53. Water access |
| 9i | Environmental enrichment | Page 53. Environmental enrichment |
| 9j | Humidity | Page 53. Humidity |
| 9k | Welfare assessment | Page 53 and 54. Welfare assessment |
| 9l | Welfare interventions | Page 53 and 54. Welfare interventions |
| 9m | Time of welfare assessment or intervention | Page 53 and 54. Time of welfare assessment or intervention |
| **10** | **Sample size** | Page 53 and 54. Sample size |
| 10a | Total number of animals used | Page 53. Total number of animals used |
| 10b | Sample size calculation | Page 53. Sample size calculation |
| 10c | Number of independent replications | Page 53 and 54. Number of independent replications |
| **11** | **Allocating animals** | Page 11, 53 and 54. Allocating animals |
| 11a | Allocation method | Page 54. Allocation method |
| 11b | Treatment and assessment of animals | Page 11, 53 and 54. Treatment and assessment of animals |
| **12** | **Experimental outcomes** | Page 11, 53 and 54. Experimental outcomes |
| **13** | **Statistical methods** | Page 12 and 54. Statistical methods |
| 13a | Details of statistical methods used | Page 12. Details of statistical methods used |
| 13b | Specify unit of analysis | Page 12. Specify unit of analysis |
| 13c | Assess normality | Page 54. Assess normality |
|  | Results |  |
| **14** | **Baseline data** | Page 16 to 18. Baseline data |
| **15** | **Numbers analysed** | Page 16 to 18.Numbers analysed |
| 15a | Animals included | Page 16 to 18. Animals included |
| 15b | Reasons for animal exclusion | No animals were excluded. |
| **16** | **Outcomes and estimation** | Page 16 to 18, Outcomes and estimation |
| **17** | **Adverse events** | No adverse events occurred. |
| 17a | Details of adverse events | No adverse events occurred. |
| 17b | Modifications to reduce adverse events | No modifications to reduce adverse events. |
|  | Discussion | Page 22 to 24, Discussion |
| **18** | **Interpretation/scientific implications** | Page 22 to 24, Interpretation/scientific implications |
| 18a | Interpretation | Page 22 and 23, Interpretation |
| 18b | Study limitations | Page 24, Study limitations. |
| 18c | Implications for 3Rs of animal use | Page 54, Implications for 3Rs of animal use |
| **19** | **Generalizability/translation** | Page 24, Generalizability/translation. |
| **20** | **Funding** | Page 24 and 25, Funding. |
