# Supplementary material for: Characterization and Comparison of Postnatal Rat Meniscus Stem Cells at Different Developmental Stages
Source: Stem Cells Transl Med. 2019 Oct 22;8(12):1318–29. doi: 10.1002/sctm.19-0125 (PMC6877772; doi:10.1002/sctm.19-0125)
Supplement: Supplementary file 8 — Appendix S1: Supplementary information [file SCT3-8-1318-s008.docx]

**Low density seeding of MeSCs**

Meniscus tissues were minced into 1–2 mm^3^ pieces. Each 100 mg tissue fragments were digested with 3 mg of collagenase type I and type II (Gibco RRL, Grand Island, NY, USA) and in 1 ml of phosphate buffered saline (PBS) at 37 ℃ for 3 hr. The suspensions were centrifuged at 1,500 g for 15 min, and the supernatant was discarded. The remaining cell pellet was cultured in low glucose DMEM (Gibco RRL, Grand Island, NY, USA) supplemented with 10% (v/v) fetal bovine serum (Gibco RRL, Grand Island, NY, USA) and 1% (w/v) penicillin-streptomycin (Gibco RRL, Grand Island, NY, USA) at 37 ℃ with 5% CO_2_.

When cells had migrated from the tissue and adhered to the culture surface, they were trypsinized and seeded at a very low density (2 cells/cm^2^) to form individual colonies. These colony-forming cells isolated from rat meniscus were designated as MeSCs. After 10–12 days, the formed colonies were trypsinized and passaged. All MeSCs in this study were of polyclonal origin. The cells were trypsinized when confluent and split 1:4. Cells that were utilized for experiments were between passages 3 and 6.

**Cell proliferation assay**

Briefly, cells from the 6 points were plated onto 96-well plates at a density of 1,000 cells/well and then cultured in standard medium for 7 days. Every day after initial plating, the medium of each well was then gently aspirated and replaced with 110 μL standard medium containing 10 μL CCK-8 solutions. After incubation in the dark with CCK-8 solution in a 5% CO_2_ incubator at 37 ℃ for 2 h. The amount of formazan produced was measured by OD values at 450nm using a spectrophotometer.

**RNA isolation and Quantitative Real-Time Polymerase Chain Reaction (qPCR) Analysis**

Total cellular mRNA was extracted by lysis in TRIZOL (Invitrogen, Carlsbad, CA, USA) followed by a one-step phenol chloroform isoamyl alcohol extraction, according to the manufacturer’s protocol. Quantitative Real-time PCR analysis of the expression of collagen II, aggrecan, Sox-9, collagen I, MKX SCX genes was carried out according to established protocols^[14, 15]^, and the results are presented as target gene expression normalized to the Glyceraldehyde 3-phosphate dehy-drogenase (GAPDH) gene. Gene expression was analyzed for fold difference using the 2^−ΔΔCT^ method. The sequences of primers used in this study are listed in Supplementary Table 1.

**Meniscectomy and MeSCs injection**

Twenty female specific pathogen free SD rats, 8 weeks old, weighing 240–260 g, purchased from the Shanghai SLAC Laboratory Aniaml Company Limited (license No. SCXK 2017-0005; Shanghai, China) were used in the *in vivo* study. Rats were grouped and housed in number of 5 per cage (size 55 cm×40 cm×20 cm) with controlled illumination (12-hour light/dark cycle), humidity(60%) and temperature (25°C). Feed and clean drinking water were provided daily, and the litter in the rat cage was replaced every 3 days to keep it clean throughout the duration of the experiment. Rats with signs of infection were excluded from the study. The sample size was calculated using an alpha of 0.05 and beta of 0.20. Experiments were designed with the 3R (replacement, refinement, and reduction of animals in research) concept in mind. Experiments involving animals have been reported according to ARRIVE guidelines.

Rats were anesthetized(intraperitoneal injection of pentobarbital, 40mg/kg), skin prepared and knee joints were opened through a medial para-patellar approach. Then the anterior half of the bilateral medial meniscus was carefully resected without cartilage and ligament injuries (Supplementary figure 2), as described previously[14, 15]. Five animals were fed within one cage and all animals were allowed free movement. All the operated animals were randomized into two groups: the MeSCs-7d-in group (n=10) and the MeSCs-7d-mix group (n=10). One and two weeks after surgery, intra-articular injection of MeSCs-7d-in or MeSCs-7d-mix (mix of MeSCs-7d-in and 50% MeSCs-7d-out at 1:1 ratio) (1×10^6^ in 100 μL PBS) was performed on the left knee, while equal volume of PBS was injected into the right knee as negative control. Rats were sacrificed after 4 weeks (n = 5 per group) and 12 weeks (n = 5 per group) of treatment. Upon sacrifice, all animals were alive and healthy without obvious adverse events, and the weights were comparable between the MeSCs-7d-in group and the MeSCs-7d-mix group both at 4 (329.2±8.1 *v.s* 332.4±11.6g, p=0.48, respectively) and 12 weeks postmeniscectomy (529.7±13.1 *v.s* 528.9±9.7g, p=0.88, respectively).
